# Experiences of Workplace Violence Among Healthcare Providers in Myanmar: A Cross-sectional Survey Study

**DOI:** 10.7759/cureus.7549

**Published:** 2020-04-05

**Authors:** Benjamin Lindquist, Michelle Feltes, Kian Niknam, Kathryn W Koval, Htoo Ohn, Jennifer Newberry, Matthew Strehlow, Rebecca Walker

**Affiliations:** 1 Emergency Medicine, Stanford University School of Medicine, Palo Alto, USA; 2 Emergency Medicine, Medical University of South Carolina, Charleston, USA; 3 Emergency Medicine, Golden Zaneka Public Co. Ltd./Parami Hospital, Yangon, MMR

**Keywords:** workplace violence

## Abstract

Background

Healthcare providers face enormous threats to personal safety from workplace violence (WPV). Prior investigations estimate a highly varied prevalence of WPV in the United States and around the world, including both verbal and physical assault. Little is known about WPV in Myanmar. Only a single prior study has evaluated WPV experiences among physicians in Myanmar, reporting an unusually low prevalence of verbal (8.7%) and physical (1.0%) assault. Given this much lower prevalence compared with similar studies in other low- and middle-income countries (LMICs), we embarked on a study to identify the prevalence of WPV in a separate cohort of healthcare providers in Myanmar.

Methods

This was a cross-sectional analysis of WPV prevalence among healthcare providers who attended a national emergency medicine conference in Myanmar in November 2018. The survey instrument was adapted from a validated survey from the Joint Program on Workplace Violence in the Healthcare Sector (International Labour Office, International Council of Nurses, World Health Organization, and Public Services International), which had been used in other global settings.

Results

Sixty-three participants completed the survey questionnaire, including 35 women (55.6%) and 26 men (41.3%). Among them, 25 (39.7%) were primary care providers. Overall, the combined prevalence of WPV in the previous 12 months was found to be 47.6% (n = 30; 95% CI: 34.9-60.6%). The prevalence of verbal assault was 47.6% (n = 30; 95% CI: 34.9-60.6%), and that of physical assault was 4.8% (n = 3; 95% CI: 1.0-13.3%). Twenty-four participants (42.4%) reported that they were encouraged to report violence in the workplace, and five (8.1%) reported they had received training on how to manage WPV. Respondents who were 30-34 years in age and those working in private facilities were significantly less likely to report WPV on univariate analysis.

Conclusion

Although our cohort comprised a limited sample of a select group of providers, we found a dramatically higher prevalence of WPV experiences among healthcare providers attending an emergency medicine conference in Myanmar when compared with a prior investigation. Very few participants had received training on WPV, and less than half reported a work culture where WPV reporting is encouraged. To combat healthcare provider shortages, more investigation is required into WPV to understand its impact and identify amelioration strategies.

## Introduction

Workplace violence (WPV) is a threat to healthcare workers globally. Whether in the form of verbal assault, physical assault, or a perceived threat of violence, WPV is a growing challenge faced by medical professionals around the world [1]. These experiences have been shown to decrease worker morale and productivity, which can lead to increased absenteeism, burnout, and attrition [2-4]. WPV and its effect on health workers place more strain on an already challenged human resource sphere in Myanmar [5]. The prevalence of WPV against healthcare groups around the world has been previously studied, revealing a wide range of reported experiences [6,7]. Only one prior study had reported WPV experiences among physicians in Myanmar, and it had revealed very low rates of both verbal assault (8.7%) and physical assault (1.0%) in the cohort studied [8]. While this study had been large (n = 196) and included respondents from public and private hospitals in three major cities, it had been performed at urban centers using an unvalidated instrument. Moreover, participants had been asked to recall episodes of WPV from patients and their families only, which may have excluded experiences of abuse from coworkers, staff, and bystanders. Similarly, providers in other settings, including acute care settings, may be at greater risk from WPV and should be included in any attempts to determine its prevalence. Since WPV rates in this study were significantly lower than those observed in most other studies of healthcare workers, it is unclear whether these low rates of WPV are translatable to other healthcare settings in Myanmar. Therefore, the aim of our study was to add to the understanding of WPV experiences in Myanmar by evaluating their prevalence among a group of healthcare providers at a national emergency medicine conference in Myanmar in November 2018 by using a well-validated instrument. We felt that If this low rate of WPV was observed in our study as well, it would indicate the need for further study to identify practices and lessons that could be useful in other settings where WPV rates are much higher.

## Materials and methods

Study design, population, and survey development

We conducted a cross-sectional study using a survey instrument adapted from a validated tool developed by the Joint Program on Workplace Violence in the Healthcare Sector (International Labour Office, International Council of Nurses, World Health Organization, and Public Services International) [9]. The survey was delivered to all healthcare professionals attending a conference on emergency care in November 2018 in Myanmar. The survey was divided into three parts: demographics, experiences related to physical assault over the previous 12 months, and experiences of verbal assault over the previous 12 months. Informed consent was obtained and participants were given written copies of the survey to complete. Participation was completely voluntary, anonymous, and without financial compensation. All responses were confidential and collected by independent research assistants for data entry. The study was approved by the Institutional Review Board of Stanford University.

Primary and secondary outcomes

The main outcomes of interest for this study were: 1) to estimate the prevalence of WPV and 2) to identify characteristics associated with increased risk of physical violence or verbal threats.

Data analysis

We used descriptive statistics to examine the distribution of outcome measures and other variables around 95% confidence intervals. Chi-squared test and Fisher exact test were used for comparing grouped data. Single variate logistic regression was used to examine measures of associations. Analyses were run using Stata 14/SE for Windows (StataCorp, College Station, TX).

## Results

A total of 63 healthcare professionals completed the study with a response rate of 63% (Abstract: Lindquist B, Feltes M, Walker R, et al. Workplace Violence Experiences Among Health Care Providers in Myanmar. Society of Academic Emergency Medicine Annual Meeting; May 15, 2019). The remaining conference attendees deferred participation. The demographic data is included in Table 1. Most participants (60.3%, n = 38) reported that there were no reporting pathways to inform employers about WPV experiences and felt that they were not encouraged to report WPV incidents (53.4%, n = 34). Further, 92.1% (n = 58) reported they had not received any training on how to manage physical abuse in the workplace.

**Table 1:** Demographic characteristics of study participants *Other includes internal medicine subspecialty physicians

Characteristics	Number of participants (%)
Age, years	
20-24	21 (33.3)
25-29	19 (30.2)
30-34	14 (22.2)
≥35	9 (14.3)
Gender	
Male	26 (41.3)
Female	35 (55.5)
Missing	2 (3.2)
Profession	
Primary care physicians	25 (39.7)
Emergency physicians	5 (7.9)
Students/house officers	15 (23.8)
Other*	15 (23.8)
Missing	3 (4.8)
Work experience, years	
<1	9 (14.3)
1-2	20 (31.8)
3-4	6 (9.5)
5-7	12 (19.0)
≥8	16 (25.4)

Prevalence of WPV

The overall prevalence of at least one experience of WPV (including both physical and verbal assault) over the previous 12 months was found to be 47.6% (n = 30; 95% CI: 34.9-60.6%). The prevalence of verbal assault (bullying, threats of physical violence, intentional use of power or harassment) was 47.6% (n = 30; 95% CI: 34.9-60.6%). The prevalence of physical assault was 4.8% (n = 3; 95% CI: 1.0-13.3%). The perpetrators of violence were mostly patients (33.3% in physical assault and 23% in verbal assault) or relatives (66.7% in physical assault and 50% in verbal assault). The remainder of perpetrators of verbal assault were: bystanders (10.3%), coworkers (31%), hospital workers (17.2%), or a group of people (13.8%).

Psychological effects

WPV or worry about potential WPV negatively affected respondents; 56% (n = 35) of the total respondents reported that they were “somewhat” or “very worried” about WPV. Of those respondents who were victims of verbal abuse, 96.7% (n = 29) reported that the experience negatively affected their psychological health (ranging from a “little bit” to “extremely”). Participants reported varying degrees of the following feelings/experiences about the incidents: repeated, disturbing memories; avoiding thinking about or talking about the abuse; being “super alert” or watchful and on guard; feeling that everything they did was a tedious effort.

Associations relating to verbal assault

Univariate analysis was only performed for verbal assault due to the very low numbers of respondents reporting physical assault. In terms of age, It was found that healthcare workers who were 30-34 years old were significantly less likely to report WPV in the survey. Regarding facility type, providers working in private facilities were significantly less likely to report WPV in the survey. Professionals who were in the first two years of their jobs were more likely to report WPV in the survey, and individuals working in urban areas were more likely to report WPV compared with those in rural areas (Table 2).

**Table 2:** Associations relating to verbal assault *Statistically significant OR: odds ratio; CI: confidence interval

Characteristics	Verbal assault experience
	OR (95% CI)
Age, years	
Reference: 20-24	
25-29	0.32 (0.09-1.18)
30-34	0.08 (0.01-0.48)*
≥35	1.75 (0.28-10.74)
Gender	
Reference: male	
Female	1.18 (0.42-3.33)
Duration of employment, years	
Reference: <1	
1-2	1.26 (0.25-6.36)
3-4	0.32 (0.04-2.62)
5-7	0.40 (0.07-2.37)
8-10	0.60 (0.08-4.40)
Environment	
Reference: urban	
Rural	0.39 (0.06-2.38)
Facility type	
Reference: public	
Private	0.27 (0.09-0.82)*

## Discussion

The objective of this study was to estimate the prevalence of WPV experiences among a sample of healthcare workers in Myanmar. We believe that this study can be an important addition to the literature on WPV experiences among healthcare workers in low- and middle-income countries (LMICs).

Compared to a previous investigation in Myanmar, we found a much higher prevalence of both verbal (47.6%) and physical assault (4.8%) experiences among our sample [8]. These results suggest that, like many other previous studies in LMICs, WPV poses a serious challenge to healthcare workers in Myanmar. These contrasting observations compared to a prior study may be explained by a number of reasons, such as the inclusion of participants who work in acute care settings, the use of a well-validated survey instrument, conducting the study in a neutral setting (i.e., not at participants' place of employment), and the inclusion of potential perpetrators of violence other than patients and family members.

Respondents between the ages of 30-34 years and those working in private facilities were significantly less likely to report a verbal assault. Over 55% of respondents reported being worried about WPV, and 62.3% reported lacking a procedure/framework for reporting concerns. Additionally, most respondents reported a lack of training on how to manage abuse at work, which highlights the importance of the implementation of administrative procedures and training.

In our survey responses, many verbal assaults were related to claims about care received, which the provider perceived as a threat to their job security. Follow-up studies may be useful to investigate the perception of job security as a predictor of decreased reporting of WPV or provider attrition. Since previous studies in other settings have shown that WPV is associated with burnout and turnover intention, it is a matter of concern that healthcare workers in Myanmar are likely similarly affected [2].

There were several limitations to this study. Firstly, the sample size was relatively small. Next, there was potential for recall bias as participants were asked to remember events that had occurred over the previous 12-month period. There was also a potential for selection bias, as the participants were self-presenting to a national conference on emergency care and may not be representative of all healthcare providers in Myanmar. Respondents attending a conference on emergency care may be more likely to treat patient populations with a higher incidence of trauma, psychiatric disease, or delirium, which may expose them to experiences of WPV on a much larger scale compared to other healthcare workers. Further, the survey required self-reporting of incidents, and we suspect that many episodes of WPV are often unreported at the time of the event and also under-reported in cross-sectional survey studies.

## Conclusions

The prevalence of WPV against healthcare workers in Myanmar, as revealed by our study among a cohort of acute care physicians, was significantly higher than previously reported in the country. In fact, it is at rates consistent with other LMICs. Mitigating the negative impacts of WPV and the threat of verbal and physical abuse is a particularly important aspect to consider in public-health workforce planning. Equipping individuals and healthcare workplaces with proper training and measures to address and prevent threats of WPV is needed to improve both health-worker safety as well as their perception of workplace safety.
